# From stress to strength: mechanotransducing poly(aneu)ploidy into a community-level advantage in cancer

**DOI:** 10.1007/s10555-026-10330-5

**Published:** 2026-03-25

**Authors:** Víctor Herrera-Fernández, Paul Dremsek, Markus Hengstschläger, Alexis J. Lomakin

**Affiliations:** 1 Center for Pathobiochemistry & Genetics, Institute of Medical Chemistry and Pathobiochemistry, Medical University of Vienna, Vienna, Austria; 2 Center for Pathobiochemistry & Genetics, Institute of Medical Genetics, Medical University of Vienna, Vienna, Austria

**Keywords:** Poly(aneu)ploidy, Cancer, Phenotypic adaptation, Mechanobiology, Cell signaling, Cellular metabolism

## Abstract

Poly(aneu)ploidy is a potent source of cellular stress that typically leads to loss of fitness, premature aging/senescence, and cell death. Yet in some systems, most notably microbial pathogens and human cancer cells under poly(aneu)ploidogenic stress, cells can adaptively remodel their phenotype, resist damage, and even convert this stress into a selective advantage. This mini-review examines current knowledge on the mechanisms underlying these adaptive responses in cancer cell communities and how poly(aneu)ploid subpopulations reshape the behavior of the entire population. Because poly(aneu)ploidy is almost invariably coupled to changes in cell size and morphology, we place particular emphasis on biophysical and mechanobiological adaptations. These include physico-chemical reprogramming, proteome remodeling, volume gain, membrane stretching, altered endocytosis, community-level metabolic rewiring, engagement of the nucleus as a key mechanosensor, and the role of mechanoreceptor channels. Finally, we discuss emerging therapeutic strategies that seek to exploit the specific vulnerabilities of poly(aneu)ploid cells. Together, these insights highlight the central role of poly(aneu)ploidy in enabling tumor adaptation and evolution, and point to new avenues for understanding cancer cell biology and designing future treatment strategies.

## Introduction: Poly(aneu)ploidy

**Fig. 1:**
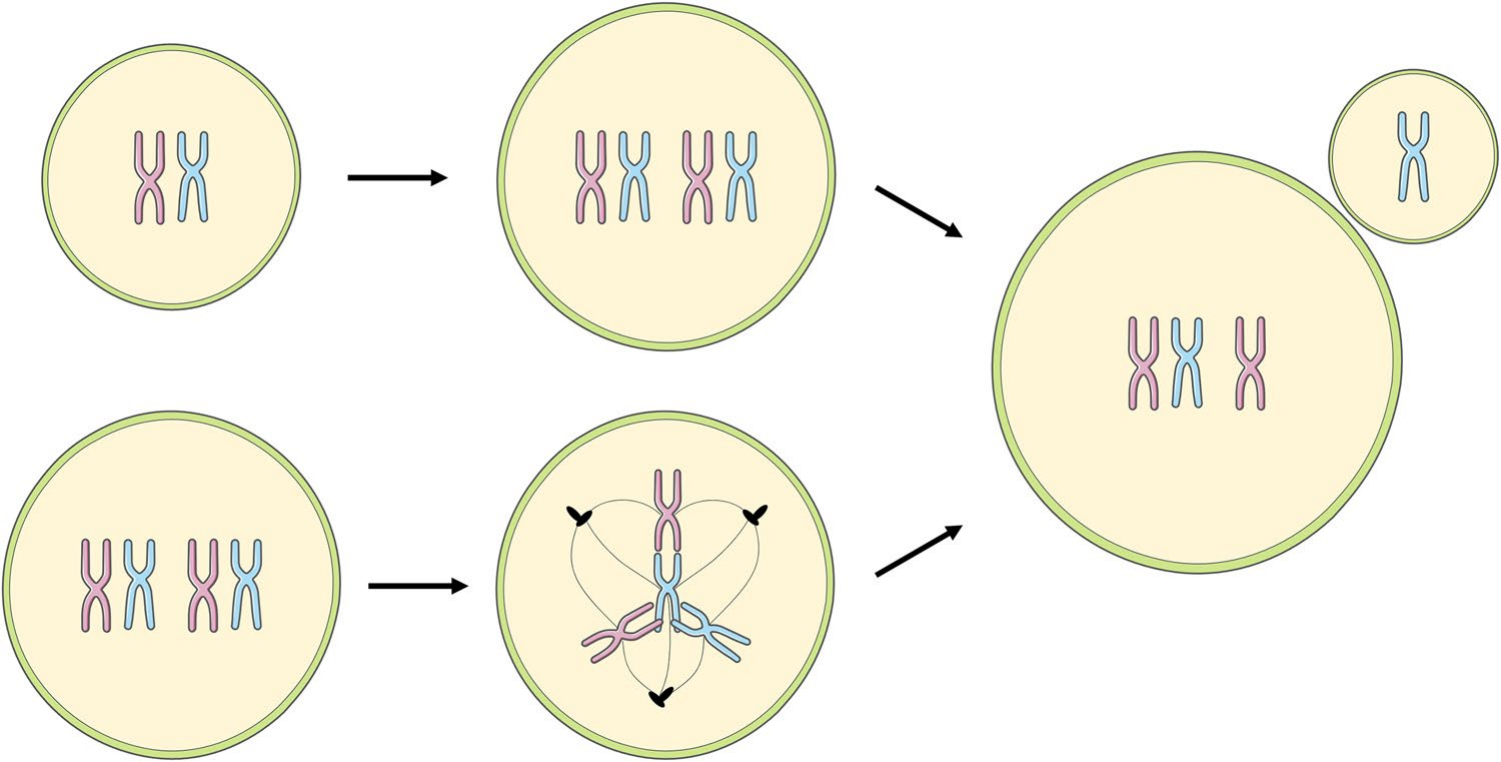
Generation of poly(aneu)ploid cancer cells. (Top row) The process begins with a normoploid (e.g., 2n) cancer cell. Through events like endoreplication, cell fusion, or failure of cytokinesis, the cell can double its chromosomal count, becoming a polyploid cell (e.g., 4n). This unscheduled polyploidization is inherently error-prone, often resulting in giant poly(aneu)ploid cells. (Bottom row) Polyploid cancer cells are susceptible to performing multipolar mitosis, a pathological event characterized by the formation of more than two mitotic poles (e.g., tripolar mitosis shown). This highly error-prone division leads to an unequal segregation of chromosomes, resulting in a highly variable progeny with an abnormal number of chromosomes (aneuploidy), further driving tumor heterogeneity

Very much like eukaryotic microbial pathogens under environmental stress [1], human tumor cells exposed to sublethal stresses within the host body often undergo so-called unscheduled polyploidization [2]. Unlike programmed somatic polyploidy, which drives terminal differentiation of cells in certain tissues during developmental processes, unscheduled polyploidization is an acute event triggered by environmental stresses, such as drug-mediated perturbations. This leads to intracellular stress because unscheduled polyploidy is intrinsically error-prone, unstable, and ultimately culminates in aneuploidy [3], defined as chromosomal stoichiometric imbalance (Fig. 1). Aneuploidy is highly stressful to cells [4] and has traditionally been associated with cellular fitness decline, premature aging/senescence, and reproductive problems.

Importantly, while most molecular traits cluster by a tumor’s tissue of origin, aneuploidy serves as a near-universal yet stochastic driver of heterogeneity, appearing in over 90% of solid tumors [5, 6]. Integrative pan-cancer analyses reveal that while both aneuploidy burden and whole-genome doubling vary widely, they represent a unique dimension of genomic chaos that does not align with standard cancer type classifications—defined by histology, tissue type, or anatomic cell of origin [7].

The etiology of this aneuploidy is rooted in a self-perpetuating cycle: mitotic errors and chromosome mis-segregation lead to the formation of micronuclei, which are prone to rupture and further DNA damage [2]. In healthy tissue, these aberrations can trigger senescence or apoptosis [8]. However, in most cancers, impaired cell cycle checkpoints—such as p53 dysfunction—act as a permissive gateway [9]. Beyond simple mis-segregation, this lack of cell cycle control facilitates chromothripsis in a subset of malignancies (2–3% of all cancers and ~25% of bone tumors), a catastrophic event where chromosomes undergo massive fragmentation and stochastic reassembly [10]. Rather than a gradual accumulation of mutations, this single-step genomic crisis can simultaneously delete tumor suppressors and amplify oncogenic drivers, such as *DYRK1A* in myeloid malignancies [11], or bypass the canonical *KRAS*-*CDKN2A*-*TP53*-*SMAD4* mutation sequence in pancreatic cancer [12]. Ultimately, these combined mechanisms allow the resulting aneuploid and polyploid progeny to survive and proliferate, establishing the highly diverse sub-populations that define aggressive malignancies [2, 8].

Despite the inherent proteotoxic stress of these states, the clinical prevalence of aneuploidy in deadly tumors [13, 14], human fungal pathogens [15, 16], and stable natural isolates of *S. cerevisiae* [17] suggests that biological cells are capable of evolving adaptations to stressful poly(aneu)ploidy. Paradoxically, such adaptations can improve the longevity of these cells and, in the long run, confer a fitness advantage to the entire pathogenic population.

What mechanisms are in place to enable these adaptive functionalities? Relevant to this question is the notion that poly(aneu)ploids often represent a lower-abundance subpopulation within a phenotypically and karyotypically heterogeneous cell community—how, then, can they impact the entire population?

## Genetic and phenotypic adaptations in poly(aneu)ploidy

The adaptive value of poly(aneu)ploidy is derived from two conceptually distinct but often synergistic mechanisms: chromosome-specific genetic dosage effects and chromosome-agnostic phenotypic rewiring. In the high-stakes environment of tumor evolution, chromosomal alterations are subject to an accelerated, punctuated form of Darwinian selection [18]. Rather than relying on the slow accumulation of point mutations, cancer cells utilize aneuploidy to achieve massive, single-step shifts in gene dosage. This allows for the rapid selection of specific chromosome gains that provide immediate fitness benefits or losses that eliminate disadvantageous regulatory constraints, often in response to specific environmental pressures [19].

A striking example of this selective efficiency is the frequent loss of the q arm of chromosome 1, an event observed in approximately 25% of all cancers [20]. This large-scale deletion often serves to phenocopy the loss of p53 signaling, functionally reinforcing the permissive gateway of checkpoint dysfunction established early in tumorigenesis. By physically removing genomic regions containing key tumor-suppressive regulators, the cell bypasses the requirement for multiple discrete mutations. Conversely, the gain of specific chromosomes can act as a dosage-dependent driver of malignancy; for instance, experimental models have demonstrated that the targeted gain of chromosome 5 significantly increases cellular migratory capacity and invasive potential in a human colon cancer cell line [21]. Similarly, in some myeloid malignancies, the amplification of the 21q region has been identified as a critical evolutionary shortcut to overexpress *DYRK1A*, a kinase that acts as a central node for proliferation and therapeutic resistance [11]. Taken together, these dosage effects provide a versatile toolkit for adaptation, as seen in MIA PaCa-2 pancreatic cancer models, where distinct chromosome copy-number profiles allow different clones within the same population to achieve divergent, yet equally fit, morphological and transcriptomic states [22].

Beyond these specific genetic identities, the state of genomic imbalance triggers a broader “aneuploidy stress response” [23]. This systemic rewiring provides a genomic buffer that facilitates rapid adaptation and therapy resistance. By doubling the available genomic material or altering the total DNA content, polyploid cells can better tolerate the deleterious effects of ongoing chromosomal instability (CIN). This stoichiometric reserve allows cells to explore emergent, non-genetic phenotypes—such as cellular dormancy—to survive lethal environments [17, 23, 24], setting the stage for the more profound physico-chemical transformations driven by proteomic imbalance.

## From proteome remodeling to physical chemistry of the cell

One emerging subgroup of phenotypic mechanisms underlying poly(aneu)ploidy tolerance involves physico-chemical alterations and mechanochemical signaling triggered by the transition to poly(aneu)ploidy. Proteome imbalance downstream of poly(aneu)ploidy [25] has been proposed to exert a non-intuitive effect on intracellular osmotic pressure [26]. In the euploid state, components of heterotypic protein complexes are expressed at levels that ensure a relatively balanced supply of subunits assembling into the same molecular complexes. Due to its dosage effect on part of, but not the entire, genome, aneuploidy disrupts this stoichiometric balance, leading to a proteome imbalance and an increase in the number of unbound proteins.

**Fig. 2:**
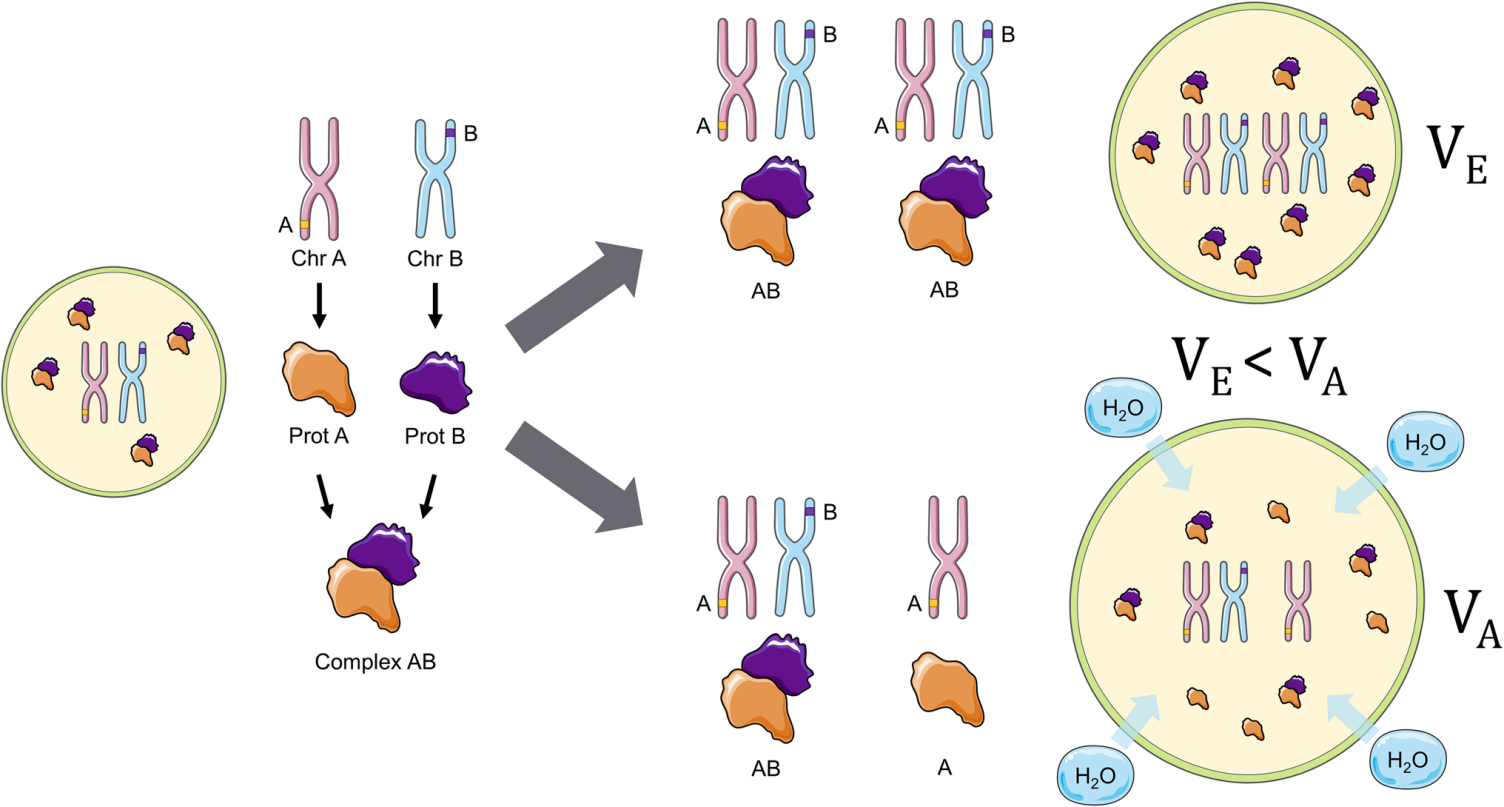
Volume regulation through aneuploidy. In a normoploid cell (left), protein subunits A (encoded by Chromosome *A*, Chr *A*) and B (encoded by Chromosome *B*, Chr *B*) are produced in balanced amounts and efficiently assemble into the functional Complex AB. In an euploid cell with increased ploidy, such as the tetraploid scenario (top right), the copy number of both Chr *A* and Chr *B* is doubled. Consequently, the production of subunits A and B remains proportional, allowing for complete assembly and leaving no orphaned subunits. The resulting equilibrium volume (V_E_) is maintained. However, in an aneuploid scenario (bottom right), the copy numbers of Chr *A* and Chr *B* are unequal (e.g., Chr *A* is duplicated but Chr *B* is not). This imbalance leads to a disproportionate increase in protein subunits, resulting in a pool of orphaned subunits (e.g., free subunit A). These excess, unassembled proteins act as osmolytes, increasing the cellular osmotic potential. This imbalance drives water (H_2_O) influx, which raises the cell’s turgor pressure and results in a significantly increased cell volume (V_A_), where V_E_ < V_A_

To manage this surplus of “orphaned” proteins and maintain essential cellular functions, cells engage active resilience mechanisms; notably, p62-dependent selective autophagy is utilized to clear these aggregated or misfolded subunits, providing a critical filter for proteomic noise [25]. However, when these compensatory pathways are overwhelmed, the accumulation of such “orphaned” proteins, acting as solutes in the cytoplasm, lowers the water chemical potential, thereby driving water influx, raising turgor pressure, and increasing cell volume [26]. Consequently, one of the most stereotypic and universal manifestations of poly(aneu)ploidy is cell swelling through cytoplasmic volume gain (Fig. 2). This model provides an elegant biophysical framework explaining how cells can enlarge independently of the canonical biosynthetic chain (DNA → RNA → Protein → Cell Mass/Volume).

Although the model represents a major conceptual advance in understanding cell size regulation, it initially failed to gain momentum due to a controversy [24] surrounding the transcriptomic signature proposed by its authors—the Common_Aneuploidy_Gene-expression (CAGE) signature [26]. A competing model suggested instead that aneuploidy induces the Environmental_Stress_Response (ESR) signature, characterized by decreased single-cell density (cell mass over volume) driven primarily by selective ribosome loss (one of the largest single contributors to cellular dry mass) rather than osmotic volume gain [24].

More recent studies employing advanced quantitative multi-omics approaches have reconciled these views, showing that both the ESR and CAGE transcriptomic signatures can coexist in aneuploid cells, albeit with variable prevalence depending on the specific aneuploidy context, and that these transcriptional patterns are not necessarily reflected at the proteome level. This historical perspective highlights the need to investigate the biology and physics of aneuploid cells beyond genes and transcripts and brings the CAGE model [26] back into focus. However, that model treats free proteins as the primary osmolytes, overlooking the dominant role of small ions and metabolites in osmotic regulation [27], and assumes ideal-solution behavior of macromolecules. These simplifications render the predicted swelling mechanism only partially plausible under realistic cellular physico-chemical conditions, emphasizing the need for further refinement and experimental testing of this model.

## The Titan phenotype: from volume gain to physical force

The acquisition of an enlarged cell phenotype driven by poly(aneu)ploidy in some human fungal pathogens is known as *titanization*, while the phenotype itself is referred to as “*Titan cells*” [28]. Along with a dramatic increase in size, fungal *Titan* cells undergo cell wall expansion and stiffening, making it harder for immune cells in the host body to engulf and digest such large and mechanically rigid objects [28]. Cell volumetric expansion and surface modifications thus enable immune avoidance within the host. This remarkable example illustrates that cell size gain and the associated biophysical processes can confer adaptive advantages to pathogenic cells.

**Fig. 3:**
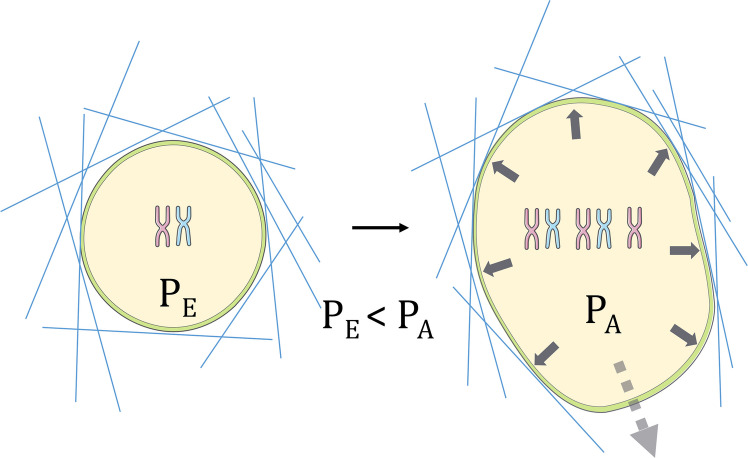
Poly(aneu)ploidy drives cell migration by increasing intracellular pressure. Poly(aneu)ploid cells (right) exhibit greater migratory potential compared to their euploid counterparts (left). As depicted, the volume gain associated with poly(aneu)ploidy (resulting from the mechanisms shown in Fig. 2) leads to a significantly greater exerted intracellular pressure (P_A_) compared to the pressure in euploid cells (P_E_), where P_E_ < P_A_. This increased turgor pressure provides the physical force required to remodel or breach surrounding extracellular matrix (ECM) barriers (represented by the blue lines). This mechanism facilitates collective invasion of new environments, enhancing the cell's ability to migrate through stiff, challenging environments and ultimately increasing tumor metastatic potential

When it comes to mammalian cancer cells, in the simplest case, poly(aneu)ploidy-associated cell swelling can contribute to the development of physical forces required for cell migration, especially in contexts where cells must push their way through confined environments, such as breaches in the basement membrane, during extravasation, or while maneuvering within interstitial spaces (Fig. 3). Indeed, MDA-MB-231 breast cancer cells experimentally enriched for polyploidy via cell sorting exhibit enhanced extravasation potential in a microfluidic system designed to reproduce the *in vivo* microvascular environment [29]. A similar trend is observed in PC3 prostate cancer cells, which display poly(aneu)ploidy and increased size in response to sublethal chemotherapeutic stress [30]. Interestingly, cell volume gain has been shown to contribute to force generation by breast epithelial cancer cells, allowing them to stretch and physically breach the stiffened basement membrane, ultimately enabling collective invasion into the stroma [31].

Thus, poly(aneu)ploidy-driven cell volume gain and expansion against external confines may represent the simplest form of mechanotransduction, enabling collective invasion and exploration of new territories, thereby benefiting the entire cancer cell population. While this scenario is highly plausible, quantitative experiments demonstrating the causal relationships between individual-cell poly(aneu)ploidy, volume gain, and force generation required to breach barriers and collectively invade new environments are still lacking. Nevertheless, MDA-MB-231 and MCF-7 breast cancer cells grown within hydrogel-based microcapsules that mimic the growth of breast tumor cells within acini surrounded by a stiff basement membrane often collectively burst out of the microcapsule, with a subset of cells exhibiting a large-sized polyploid phenotype [32], suggesting that the conjecture linking poly(aneu)ploidy, cell volume gain, and hydraulic force generation may indeed be a viable hypothesis.

## Membrane stretch, endocytosis, and community metabolism

The cell swelling that accompanies poly(aneu)ploidy acquisition is known to induce plasma membrane unfolding and stretch, which physically opposes the membrane’s ability to curve inward, thereby impairing endocytosis [26]. A crucial functional consequence of this mechanically impaired endocytosis is increased retention of plasma membrane-associated nutrient transporters that are normally dynamically recycled via endocytosis. Since the transporters remain on the plasma membrane for a longer period, this results in a higher steady-state concentration of transporters and, consequently, a higher overall rate of nutrient influx [26].

In a phenotypically diverse cell population, this can lead to a situation where more normoploid neighboring cells begin to suffer: those relying on the same external nutrient pool become nutrient-starved, leading to ecological competition and selection, a factor highly critical for the reproductive success of a pathogenic cell population. At the same time, some plasma membrane transporters are reversible (uniporters, exchangers, facilitated diffusion carriers). In such cases, a higher transporter number retained at the plasma membrane can increase both influx and efflux of nutrients/metabolites, depending on the concentration gradient.

**Fig. 4:**
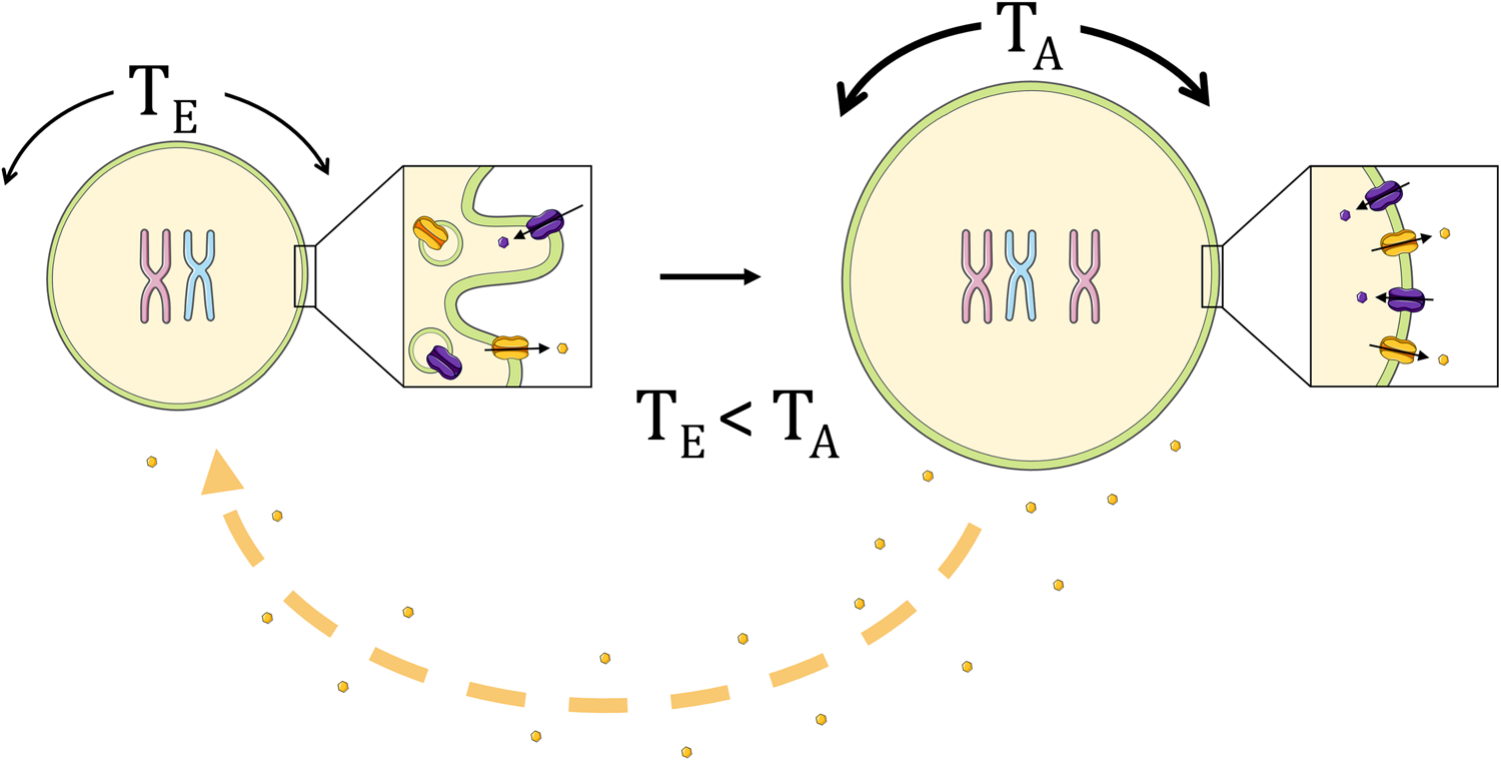
Community metabolism between cancer subpopulations. The cell swelling and subsequent volume increase in aneuploid cells result in a significantly increased plasma membrane tension (T_A_) compared to the tension in their euploid counterparts (T_E_), where T_E_ < T_A_. This elevated membrane tension impedes membrane curvature, leading to impaired endocytosis. Conversely, this favors retention and thus activity of importers and exporters at the plasma membrane, which modulates the overall rate of nutrient influx and efflux. This process would enable poly(aneu)ploid cancer cells to serve as “helper” cells for the tumor community in two ways: by exporting excess nutrients or metabolic byproducts into the extracellular medium, and by secreting pro-tumorogenic paracrine factors, potentially as a product of the senescence-associated secretory phenotype (SASP)

This may lead to a nuanced situation, particularly when the external environment becomes nutrient-depleted and poly(aneu)ploid cells with a high rate of nutrient influx fail to privatize these resources due to altered metabolism. In such scenarios, poly(aneu)ploid cells can increase efflux, effectively converting into “feeder” cells that support the rest of the population (Fig. 4). The senescence-associated secretory phenotype (SASP) of poly(aneu)ploids [33–35] can be considered a form of such metabolic cooperation. Moreover, the emergence of SASP in poly(aneu)ploid cancer cells leads to the secretion of paracrine pro-tumorigenic effectors, such as invasion, migration, angiogenesis, and inflammation modulators [36, 37]. This fact reinforces the idea of a role of these cells as “helpers” for the rest of the population. The reliance of these cell-nonautonomous mechanisms on the mechanobiology of poly(aneu)ploids requires in-depth mechanistic investigation.

Additionally, the model of mechanically impaired endocytosis due to cell volume gain upon poly(aneu)ploidization appears to be contradicted by the recent discovery that, contrary to the common dogma that cell volume inflation automatically translates into a decreased surface area-to-volume (SA/V) ratio, polyploid cancer cells somehow sense the state of poly(aneu)ploidization and excessive growth, scaling their plasma membrane material approximately isometrically with cell size to ultimately maintain a constant SA/V ratio [38]. This, in turn, requires that the plasma membrane accumulates more folds as cell size increases, effectively canceling the proposed plasma membrane unfolding-and-stretch scenario.

However, one must consider that the increased total plasma membrane material and its wrinkling are most likely long-term adaptations driven by metabolic rewiring and enhanced lipid biosynthesis. In contrast, the initial cell volume gain upon poly(aneu)ploidy and the accompanying plasma membrane unfolding and stretch may represent a more acute response operating on timescales shorter than those required for metabolic or signaling rewiring to produce additional membrane material. Yet another explanation is that published cases of ploidy gain in cancer cells involve true polyploidy (2 × 2 = 4 or 4 × 2 = 8), which may employ mechanisms distinct from those observed during aneuploidy. We consider this unlikely, given that almost all established human cancer cell lines and most patient-derived tumors exhibit considerable aneuploidy for specific chromosomes at steady state [5, 6], which becomes dramatically amplified and randomized during events triggering unscheduled whole-genome doubling through abnormal mitosis [13]. Furthermore, even in genetically stable systems other than cancer, already true tetraploid cells are most susceptible to undergo chromosomal instability and aneuploidy due to a disbalanced scaling between DNA content and DNA replication factors, leading to DNA damage and an aberrant S phase [3, 39].

Nonetheless, further investigation of these fascinating apparent contradictions and the regulatory logic underlying them represents an exciting direction for future research. Combining phenotypic analysis of single cells with more precise measurements of their ploidy and chromosomal stoichiometry will likely be of great value. An example of this is the recently introduced MAGIC technique, a platform coupling automated microscopy with targeted photolabelling and single-cell genomics to gain insights into chromosomal abnormalities from studying nuclear atypia [40].

## The nucleus as a mechanosensor of poly(aneu)ploidy

Given that the nucleus has recently emerged as a critical mechanosensor [41, 42] and that it hosts the extra chromosomes acquired during poly(aneu)ploidization, this organelle is an excellent candidate for such sensing. Indeed, the nucleus of cells harboring extra chromosomes undergoes both volumetric and surface area expansion [43]. Recent work further confirms that this nuclear expansion is a hallmark of aneuploid states, as cells harboring extra chromosomes consistently exhibit significant increases in both nuclear volume and surface area [44].

On one hand, this increase in nuclear size might reflect an adaptation to cytoplasmic volume gain, maintaining a constant nuclear-to-cytoplasm ratio [45]. On the other hand, experimental evidence from acutely induced aneuploidy demonstrates that these karyotypic changes are directly sensed by the nucleus, triggering rapid nuclear envelope (NE) remodeling [44, 46]. Specifically, using a tunable system of chromosome mis-segregation, it has been shown that mitotic errors trigger acute nuclear deformation, nuclear softening, and significant alterations to the lamin and heterochromatin architecture [46]. The fact that these induced errors provoke such immediate remodeling suggests that the nucleus mounts a robust, active mechanosensitive response to aneuploidy.

Furthermore, nuclei isolated from cells bearing extra chromosomes maintain their increased size relative to normoploid nuclei even after reconstitution of sucrose density–purified nuclei in PBS [43]. This suggests that nuclear expansion either occurs independently of cytoplasmic volume gain or is a consequence of it but is strongly reinforced at the nuclear level to an irreversible degree.

A crucial consequence of this nuclear expansion phenotype in cells with extra chromosomes is that the shape of their nuclei becomes highly distorted or deformed, independent of chromosomal identity [43, 46]. The same trend appears when looking at human dermal fibroblasts carrying clinically relevant trisomies 21, 18, and 13. These clinical data underscore that, no matter which specific chromosome is gained, the observable physical phenotype remains fundamentally the same [43].

An intuitive mechanism for this is that, in order to accommodate extra chromosomes, poly(aneu)ploid nuclei must increase their internal volume as well as the surface area (NE and lamina) available for interactions with the growing number of heterochromatic regions provided by additional chromosomes, which in turn can lead to nuclear shape distortions. Given that cells harboring yeast artificial chromosomes (YACs) containing heterochromatic human DNA do not show considerable changes in nuclear size or shape [43], it is unlikely that extra chromosomes *per se* provoke nuclear remodeling. The latter is most likely driven by the dramatic rewiring of cellular metabolism and shifts in lipid synthesis predicted to occur in poly(aneu)ploids [47]. Indeed, it has been shown that remodeling of lipid metabolism and the under- or overproduction of specific lipid species enriched in nuclear membranes determine the size and shape of aneuploid nuclei with extra chromosomes [43].

This remodeling manifests as localized alterations in the nuclear lamina, which becomes patchy, with thin regions interspersed among thicker ones [43]. Such a patchy structure can predispose the nuclear membrane near weak, thin regions of the lamina to detach, bulge out, and stretch. Nuclear membrane stretch in this context has been shown to be sensed by mTORC2 [46], previously identified as a plasma membrane tension sensor [48]. At the same time, mTORC2 and its plasma membrane stretch-sensing capacity are crucial for persistent cell migration under environmental confinement [49].

Thus, one can speculate that nuclear membrane stretch sensing via mTORC2, and the signaling cascades downstream of it, may enable or reinforce aneuploid cell migration and facilitate dissemination of karyotypic diversity to distant sites. Although it remains to be investigated why and how mTORC2 becomes engaged as a nuclear membrane tension sensor, rather than other molecular sensors, in the context of aneuploidy, and how broadly applicable this mechanotransduction pathway is, these findings illustrate that poly(aneu)ploidy is not merely a chromosomal stoichiometry aberration but also a biophysical process that can be sensed and converted into molecular signals controlling complex cell behaviors.

## Routing in and out of poly(aneu)ploidy by engaging molecular mechanosensors

Unscheduled polyploidy in cancer cells is a highly unstable state [44], and in the majority of studied cases, cells in this state tend to engage mechanisms for depolyploidization. For example, they can undergo neosis, a depolyploidization program characterized by production of aneuploid daughter cells via nuclear budding and asymmetric cytokinesis, involving mitotic and meiotic genes. This mechanism is used by tumor cells to depolyploidize after radiotherapy or chemotherapy to promote tumor re-emergence [50, 51]. Quite often, cancer cells also activate programs that prevent the emergence of polyploidy, frequently utilizing the same conserved cellular and molecular machinery to achieve both strategies.

This regulation appears to be orchestrated by a mechanosensitive safeguard system that may span from the cell cortex to the nuclear interior. At the plasma membrane, the mechanosensitive Ca^2+^ channel PIEZO1 acts as a primary sensor of global cell tension; tuning its activity is crucial for triggering premature centriole disengagement, resulting in supernumerary centrosomes [52]. The formation of supernumerary centrosomes enables cells to enter a state of multipolar mitosis, which in turn serves as an efficient mechanism of depolyploidization in tetraploid tumor cells [53]. At the same time, PIEZO1 inhibition or deletion in otherwise non-polyploid cancer cells leads to cytokinetic failure, producing large polyploid cells [54]. It appears that the localized force generated during abscission activates PIEZO1 and enables localized Ca^2+^ entry, which is crucial for the activation of the ESCRT-III-based machinery required to finalize the physical separation of cells during the final stages of cytokinesis.

While the cortical role of PIEZO1 is well-documented, the extreme nuclear deformation and curvature characteristic of the “meiotic-like” budding phase in neosis suggests the existence of a parallel mechanosensing event at the NE. A compelling candidate for this intranuclear regulation is cPLA2 (Cytosolic Phospholipase A2), a protein recently identified as a dedicated sensor for NE tension [42]. It is plausible that the physical strain placed on the NE during neotic genomic sorting triggers cPLA2 translocation to the nuclear membrane in response to lipid packing stress. In this conceptual model, cPLA2 would serve as the internal counterpart to PIEZO1, providing the structural feedback necessary to accommodate drastic nuclear remodeling. By modulating membrane plasticity—and potentially coordinating with the same ESCRT-III machinery utilized at the cortex—cPLA2-mediated sensing may represent a specialized adaptation to prevent catastrophic nuclear rupture during the neotic life cycle, thereby ensuring the survival of aneuploid progeny.

## Therapeutic strategies targeting the cancer poly(aneu)ploid phenotype

The unique, mutation-independent, systems-level adaptations of poly(aneu)ploid cancer cells offer several distinct therapeutic liabilities exploitable through precision-phenotype approaches. These strategies aim for synthetic lethality by targeting the compensatory mechanisms these cells must rely upon to survive the chronic stress imposed by their genomic and physical instability.

The high proteotoxic burden associated with aneuploidy can be addressed by exploiting the required increase in RNA and protein degradation. For instance, bortezomib elicits a synthetic lethal interaction by inhibiting the proteasome in highly aneuploid cancers, such as multiple myeloma, though success in solid tumor trials remains limited [44, 55]. Similarly, the intrinsic mitotic defects of aneuploid cells can be aggravated by mitotic checkpoint inhibitors (e.g., KIF18A inhibitors), selectively reducing cell fitness by enhancing the existing mitotic dysfunction [56].

Furthermore, the metabolic and cytoskeletal reorganization observed in poly(aneu)ploid cells is a key target. The mTOR signaling pathway is a central focus of research, with numerous clinical trials investigating rapamycin derivatives or next-generation pan-mTOR inhibitors [57]. However, an alternative strategy could be the specific targeting of mTORC2 to dismantle the poly(aneu)ploid cell’s anabolic and pro-motility machinery. Given the structural similarities between the two complexes, this selectivity is challenging to achieve via small molecules. Nonetheless, this concept has been validated pre-clinically, where nanoparticle-delivered RNAi targeting the mTORC2-specific subunit Rictor has shown promising effects in breast cancer models [58]. Conversely, the polyploid precursor state is vulnerable to AMPK activators like resveratrol and salicylate (aspirin’s prodrug, which also inhibits mTOR signaling [59]), which selectively eliminate these cells by inducing metabolic strain [60].

Finally, the massive size and migratory requirements of these cells necessitate precise mechano-osmotic homeostasis, creating a biophysical weakness. Mechanoreceptor channels, such as TRPV4 and TRPM7, critically contribute to volume and motility regulation in cancer [61, 62], while PIEZO1 activity is critical for supernumerary chromosome generation [52], suggesting that subtle modulation of these receptors could push poly(aneu)ploid cancer cells out of their required “Goldilocks zone” and induce synthetic lethality.

## Conclusion

While many aspects of poly(aneu)ploid cell mechanobiology remain to be clarified and discovered, it is increasingly evident that these non-genetic (phenotypic) mechanisms represent an exciting new frontier in cell biology and biomedical research. Poly(aneu)ploidy-driven changes in cell mechanics, osmotic balance, and metabolic organization not only reshape fundamental principles of cellular homeostasis but also redefine how cells adapt to stress, resist therapy, and modulate their microenvironment.

By coupling genomic imbalance to physico-chemical and mechanical feedback, poly(aneu)ploid cells reveal that survival and adaptation can emerge from systems-level reorganization rather than from specific gene mutations alone. Understanding how these processes integrate at the levels of the proteome, metabolome, and cell architecture will be essential for explaining the persistence of drug-tolerant and senescence-like states in cancer, as well as for uncovering general rules of cellular aging and stress resilience.

Ultimately, dissecting the mechanobiology of poly(aneu)ploid cells offers a unique opportunity to bridge molecular, biophysical, and evolutionary perspectives—illuminating how cells sense, accommodate, and exploit genome imbalance to survive and evolve under adverse conditions.

## Data Availability

No datasets were generated or analysed during the current study.
